# Prognostic impact of *NF1* mutation in Korean cohort with glioblastoma

**DOI:** 10.3389/fneur.2026.1752936

**Published:** 2026-05-21

**Authors:** Ryong Heo, Yeosong Kim, Sehyeon Kim, Boseong Kim, Sinsoo Jeun, Chihyun Park

**Affiliations:** 1 Department of Data Science, Kangwon National University, Chuncheon-si, Gangwon-do, Republic of Korea; 2 Department of Neurosurgery, GSAM Hospital, Gunpo, Republic of Korea; 3 Department of Biotechnology and Bioengineering, Kangwon National University, Chuncheon-si, Gangwon-do, Republic of Korea; 4 Department of Neurosurgery, Seoul St. Mary's Hospital, College of Medicine, The Catholic University of Korea, Seoul, Republic of Korea; 5 Department of Computer Science and Engineering, Kangwon National University, Chuncheon-si, Gangwon-do, Republic of Korea

**Keywords:** glioblastoma, Korean glioblastoma patient cohort, *NF1* mutation, O6-Methylguanine-DNA methyltransferase promoter unmethylation, prognostic markers in GBM

## Abstract

**Background:**

Glioblastoma (GBM) is a highly malignant brain tumor with a poor prognosis. Despite advancements in treatment, identifying robust biomarkers remains a challenge for improving clinical management. This study aims to identify poor prognostic factors and gain insights into their clinical significance through the analysis of next generation sequencing (NGS) data from GBM patients in a Korean cohort.

**Methods:**

An Overall Survival (OS) analysis and various statistical assessments were conducted on 95 GBM patients using data obtained from the Oncomine comprehensive assay.

**Results:**

A demographic and clinical analysis of the 95 GBM patients, grouped by survival duration, revealed differences in age (*p* = 0.010), unmethylated O6-Methylguanine-DNA methyltransferase promoter (MGMTp) status (*p* = 0.043), and Blood Urea Nitrogen (BUN) levels (pre-operative *p* = 0.001, post-operative *p* = 0.003). In the analysis to identify genetic factors, 11 samples (11.5%) with *NF1* mutations were identified. Multivariable Cox analysis indicated that *NF1* mutation and unmethylated *MGMT*p status showed a potential correlation with decreased OS (*p* = 0.042 and *p* < 0.001, respectively). Kaplan-Meier OS analysis further supported these findings, as demonstrated by the log-rank test (*p* = 0.044 and *p* = 0.007, respectively).

**Conclusion:**

In this study, we observed that *NF1* mutation was associated with poorer OS in a cohort of Korean GBM patients across several analyses. Given the limited sample size (*N* = 11), these findings should be interpreted as exploratory; however, they provide hypothesis-generating evidence suggesting that *NF1* may serve as a potential complementary biomarker in assessing the prognosis of GBM patients in future clinical evaluations.

## 1 Introduction

GBM is the most common and aggressive primary brain tumor in adults, typically associated with a poor prognosis (1). Even with standard treatment, including maximal safe resection, concomitant chemoradiation, followed by adjuvant chemotherapy, the median OS remains very low, ranging from 12 to 15 months (2). In addition to standard treatment, various therapies such as vaccine therapy, CAR-T therapy, targeted therapy, and immunotherapy are being explored (3). Currently, FDA-approved targeted therapies available on the market include those targeting *VEGF, FGFR, BRAF V600E*, and *NTRK* fusion, and their use is gradually increasing worldwide (4). With the growing importance of genetic factors, NGS analysis for GBM patients is now covered by insurance in South Korea. Since 2017, South Korea has provided insurance coverage for NGS analysis for GBM patients (5). Consequently, most patients who underwent GBM surgery at our institution after 2017 received NGS analysis. NGS has made an innovative contribution to identifying genetic variations by enabling a comprehensive analysis of the molecular characteristics of GBM (6). We conducted an exploratory NGS study on a retrospective cohort of GBM patients in Korea, aiming to identify genetic factors that may influence patient survival duration. Through this preliminary analysis of the Korean cohort of GBM patients treated at Seoul St. Mary's Hospital, we identified that alterations in the *NF1* gene and the methylation status of the *MGMT*p serve as potential indicators of poorer survival outcomes in this cohort.

## 2 Methods

### 2.1 Patient selection

This study was approved by the Institutional Review Board (IRB) of our institution (OC24WIDI0124), and due to its retrospective nature, the IRB waived the requirement for informed consent. Since 2017, our two institutions have performed NGS on GBM patients. From 2017 to August 2022, a total of 96 patients were diagnosed with GBM at Seoul St. Mary's Hospital. According to the 2021 EANO and CNS WHO guidelines, 95 of these patients were identified as having GBM (7, 8). We followed the prognosis of these patients through April 2024.

### 2.2 Data collection

Clinical and molecular data were extracted from electronic medical records and NGS reports. Clinical variables included sex, age at diagnosis, and OS. Targeted panel-based NGS analysis was performed on tumor samples extracted from the patients. These samples underwent amplicon capture-based library preparation and were sequenced using the Oncomine Comprehensive Assay platform (9).

### 2.3 Molecular pathology

#### 2.3.1 IDH mutation analysis

*IDH* mutation status was determined as part of routine molecular pathology evaluation using a targeted NGS assay (Oncomine Comprehensive Assay Plus, Thermo Fisher Scientific). Sequencing was performed on the Ion S5 XL platform, and data were analyzed using standard bioinformatics pipelines (Torrent Suite, Ion Reporter, and the Oncomine knowledgebase) with alignment to the hg19 reference genome. Tumors in which no pathogenic *IDH* mutation was detected were classified as *IDH* wildtype.

#### 2.3.2 MGMT promoter methylation analysis

MGMTp methylation status was determined from pathology reports using methylation-specific real-time PCR (semi-quantitative methylation-specific PCR; SQ-MSP) performed on DNA extracted from tumor specimens, according to standard clinical interpretation criteria.

#### 2.3.3 TERT promoter mutation analysis

*TERT* promoter mutation status was evaluated as part of the molecular pathology workup using Sanger sequencing and/or a targeted NGS assay (Oncomine Comprehensive Assay Plus, Thermo Fisher Scientific). Sanger sequencing was performed to assess hotspot mutations within the *TERT* promoter region, while targeted NGS was used to detect promoter hotspot variants when sufficient material and coverage were available.

#### 2.3.4 EGFR amplification analysis

*EGFR* status was initially assessed by immunohistochemistry to evaluate protein overexpression patterns. To determine *EGFR* gene amplification, copy number variation (CNV) analysis was subsequently performed using a targeted NGS panel (Oncomine Comprehensive Assay Plus, Thermo Fisher Scientific). *EGFR* amplification was defined based on predefined CNV thresholds, and IHC results were used as supportive information, rather than as a surrogate for gene amplification.

Further details of the molecular and cytogenetic diagnostic procedures are provided in Supplementary Methods S1.

### 2.4 Statistical methods

For accurate analysis, all continuous variables were tested for normality using the Kolmogorov-Smirnov test. Variables showing a normal distribution were analyzed using Student's *t*-test, while non-normally distributed variables were assessed using the Mann-Whitney U-test to calculate *p*-values. For categorical variables, the Chi-square test or Fisher's exact test was used as appropriate. OS analysis was performed using Kaplan-Meier survival curves, and *p*-values were calculated via the log-rank test. To evaluate the potential prognostic utility of the *NF1* mutation while strictly adhering to the events-per-variable guideline and preventing statistical overfitting, a pre-specified multivariable Cox proportional hazards model was constructed. Variables were selected based on the 2021 WHO classification criteria for IDH wildtype glioblastoma. The proportional hazards assumption was verified for all covariates using the Schoenfeld residuals test. The final multivariable Cox model was adjusted for six core variables: ECOG performance status, initial surgical method (tumor resection vs. biopsy), MGMT promoter methylation, *TERT* promoter mutation, *EGFR* amplification, and *NF1* mutation status. Additionally, a formal interaction analysis between *NF1* mutation and MGMT promoter methylation status was conducted. Laboratory findings were analyzed using the mean values of pre-operative and post-operative results. For post-operative data, to ensure consistent evaluation despite varying follow-up periods, only results obtained within 60 days post-surgery were included. All statistical analyses were conducted using R software version 4.4.0, with statistical significance defined as a two-tailed *p*-value < 0.05 in all cases.

## 3 Results

### 3.1 Cohort dataset description

We obtained data from a total of 95 GBM patients who had sufficient data for this study. During the follow-up period, a total of 73 events (deaths) were observed. The age range of all 95 patients was 15 to 92 years, with a median age at diagnosis of 70. The gender ratio was 53 males to 42 females. We designated Methylated/Unmethylated MGMTp as MGMTp (+/–) and gene wildtype/mutant as (+/–) with gene symbol. Table 1 presents the demographic status and laboratory findings of the patient cohort, stratified into two groups based on the median OS duration of 14 months for GBM. In addition to demographic and molecular variables, we analyzed selected routine perioperative laboratory parameters, including BUN and glucose levels, to provide complementary clinical context alongside molecular prognostic factors, based on prior evidence suggesting their potential relevance in GBM. Laboratory values were categorized into pre-operative and post-operative measurements to account for perioperative physiological changes, and mean values with standard deviations are reported. Data from six patients without surgical records and one patient lacking post-operative laboratory results were excluded, leaving 88 patients included in the laboratory analysis. Statistically significant differences were observed for age (*p* = 0.01) and MGMTp methylation status (*p* = 0.043). Among the laboratory parameters, BUN levels showed significant differences both pre-operatively (*p* = 0.001) and post-operatively (*p* = 0.002), whereas glucose levels did not show significant associations with OS. The observed BUN findings were consistent with previous studies in GBM (10). In the NGS analysis of the entire Seoul patient cohort, the most frequent genetic mutations were *TERT* promoter (*TERT*p, *N* = 49), *TP53* (*N* = 32, 33.7%), *PIK3CA, NF1, PTEN*, and *EGFR* (all *N* = 11, 11.6%). Among amplification mutations, *EGFR* (*N* = 28, 29.4%), *PDGFRA* (N = 19, 20%), *CDK4* (*N* = 17, 17.9%), and *KIT* (*N* = 14, 14.7%) were the most commonly observed. A comparison of these mutation and amplification frequencies with those reported in previously published GBM cohorts is summarized in Table 2.

**Table 1:** Demographic table with OS of GBM patients.

Prognostic factors		Overall survival periods		*P*_value
		Under 14 months (*N* = 42)	Over 14 months (*N* = 53)	
Sex	Male	16 (38.1%)	26 (49.1%)	0.390
	Female	26 (61.9%)	27 (50.9%)	
Age	>= 65	35 (83.3%)	30 (56.6%)	**0.010**
	< 65	7 (16.7%)	23 (43.4%)	
Ki-67	>= 0.35	16 (38.1%)	22 (41.5%)	0.899
	< 0.35	26 (61.9%)	31 (58.5%)	
Unmethylated MGMTp		27 (64.3%)	23 (43.4%)	**0.043**
Laboratory findings (*N* = 88)		Under 14 months (*N* = 39)	Over 14 months (*N* = 49)	
Urea Nitrogen (mg/dl)	Preoperative	19.0 (6.2)	15.0 (4.3)	**0.001**
	Postoperative	18.3 (5.5)	15.6 (4.3)	**0.002**
Glucose (mg/dl)	Preoperative	138.1 (40.5)	122.0 (23.5)	0.168
	Postoperative	126.2 (23.3)	120.8 (20.0)	0.202

Bold values represent statistically significant results.

**Table 2:** Comparison of genetic alteration frequencies between the Seoul GBM cohort and reported GBM studies.

Gene	Mutation frequency (*N*, %)	Amplification frequency (*N*, %)	Reported frequency in GBM (%)	Reference
*TERT*p	49 (62.1%)	—	66%~80%	Lee et al. (23), Giunco et al. (24)
*TP53*	32 (33.7%)	—	15~36%	Esperante et al. (25)
*PIK3CA*	11 (11.6%)	—	3~13%	Brito et al. (26), Pandey et al. (27)
*NF1*	11 (11.6%)	—	10~14%	Brennan et al. (28), Pandey et al. (27)
*PTEN*	11 (11.6%)	—	30~40%	Benitez et al. (29)
*EGFR*	11 (11.6%)	28 (29.4%)	Mut 10%~16%, Amp 43%~57%	Pandey et al. (27), Ezzati et al. (30)
*PDGFRA*	—	19 (20.0%)	19%-29%	Carlotto et al. (31), Higa et al. (32)
*CDK4*	—	17 (17.9%)	11%~15%	Rollbrocker et al. (33), Pandey et al. (27)
*KIT*	—	14 (14.7%)	14.00%	Carlotto et al. (31)

### 3.2 Genetic and clinical factors influencing in GBM patients

To identify independent prognostic factors for OS in our cohort, we performed univariate and multivariable Cox proportional hazards analyses (Table 3). Following the verification of proportional hazards assumptions, the final multivariable model was constructed using six core clinical and molecular variables: ECOG performance status, initial surgical method, MGMT promoter methylation status, *TERT* promoter mutation, *EGFR* amplification, and *NF1* mutation. In the multivariable analysis, two factors demonstrated a statistically significant association with poorer OS: unmethylated MGMT promoter [HR: 2.70 (95% CI: 1.56–4.68), *p* < 0.001], and the presence of *NF1* mutation [HR: 2.22 (95% CI: 1.03–4.80), *p* = 0.042]. Other baseline covariates adjusted in the model did not reach statistical significance. These included initial surgical method (biopsy vs. resection) [HR: 1.32 (95% CI: 0.39–4.45), *p* = 0.659], ECOG performance status [HR: 1.17 (95% CI: 0.89–1.55), *p* = 0.259, per 1-point increase], *TERT* promoter mutation [HR: 1.12 (95% CI: 0.64–1.94), *p* = 0.698], and *EGFR* amplification [HR: 0.99 (95% CI: 0.54–1.84), *p* = 0.986]. Given that unmethylated MGMT promoter and *NF1* mutation emerged as the notable prognostic factors in this model, subsequent analyses were conducted to evaluate the combined impact and potential interactions of these two variables on patient survival.

**Table 3:** Cox analysis with genetic and clinical factors.

Variable	Univariate		Mutivariate		Nums
	95% CI	*P*-value	95% CI	*P*-value	
MGMTp status					*N* = 95
Methylated	1.00		1.00		*N* = 45
Unmethylated	1.9 (1.18–3.07)	**0.008**	2.7 (1.56–4.68)	**<** **0.001**	*N* = 50
ECOG (per 1 point increase)	1.45 (1.14–1.85)	**0.003**	1.17 (0.89–1.55)	0.259	*N* = 95
Initial surgical method					*N* = 89
Tumor resection	1.00		1.00		*N* = 85
Biopsy	1 (0.31–3.2)	0.997	1.32 (0.39–4.45)	0.659	*N* = 4
*NF1* mut	1.99 (1.01–3.95)	**0.048**	2.22 (1.03–4.8)	**0.042**	*N* = 11
*TERT*p mut	1.16 (0.71–1.9)	0.547	1.12 (0.64–1.94)	0.698	*N* = 49
*EGFR* amp	0.92 (0.54–1.56)	0.755	0.99 (0.54–1.84)	0.986	*N* = 28

Bold values represent statistically significant results.

### 3.3 Genetic and clinical associations with/without *NF1* mutation

To explore potential co-occurrence patterns between *NF1* and other clinical or genetic profiles, we conducted an exploratory association analysis across the cohort (Table 4). No significant differences in major clinical characteristics were observed between the *NF1*(+) and (–) groups. Among the evaluated genetic factors, *PPM1D* and *SETD2* mutations demonstrated a statistically significant co-occurrence with the *NF1*(–) (*p* = 0.035 and *p* < 0.001, respectively). Specifically, the occurrence of *PPM1D* and *SETD2* mutations was notably enriched in the *NF1*(–) group (*n* = 11) compared to the wildtype group (*n* = 84). However, considering the extremely low absolute number of these specific mutation events within our cohort, it is difficult to definitively assert robust statistical or clinical significance, and these exploratory findings should be interpreted with caution.

**Table 4:** Associated factors with *NF1*.

Prognostic factors		NF1 mutation (*N* = 11)	NF1 wildtype (*N* = 84)	*P*_value
Sex	Male	6 (54.5%)	47 (56%)	1.000
	Female	5 (45.5%)	37 (44%)	1.000
Age	>= 65	5 (45.5%)	25 (29.8%)	0.315
	< 65	6 (54.5%)	59 (70.2%)	0.315
Ki-67	>= 0.35	9 (81.8%)	48 (57.1%)	0.190
	< 0.35	2 (18.2%)	36 (42.9%)	0.190
Unmethylated MGMTp		5 (45.5%)	45 (53.6%)	0.612
*CDK6* amp		1 (9.09%)	3 (3.57%)	0.394
*PIK3CA* amp		2 (18.18%)	2 (2.38%)	0.065
*SOX2* amp		0 (0%)	2 (2.38%)	1.000
*RHEB* amp		1 (9.09%)	1 (1.19%)	0.219
*PPM1D* mut		2 (18.18%)	1 (1.19%)	**0.035**
*SETD2* mut		3 (27.27%)	0 (0%)	**0.001**
*PIK3CA* mut		3 (27.27%)	8 (9.52%)	0.114

Bold values represent statistically significant results.

### 3.4 OS outcomes according to MGMTp and *NF1* status

**Figure 1:**
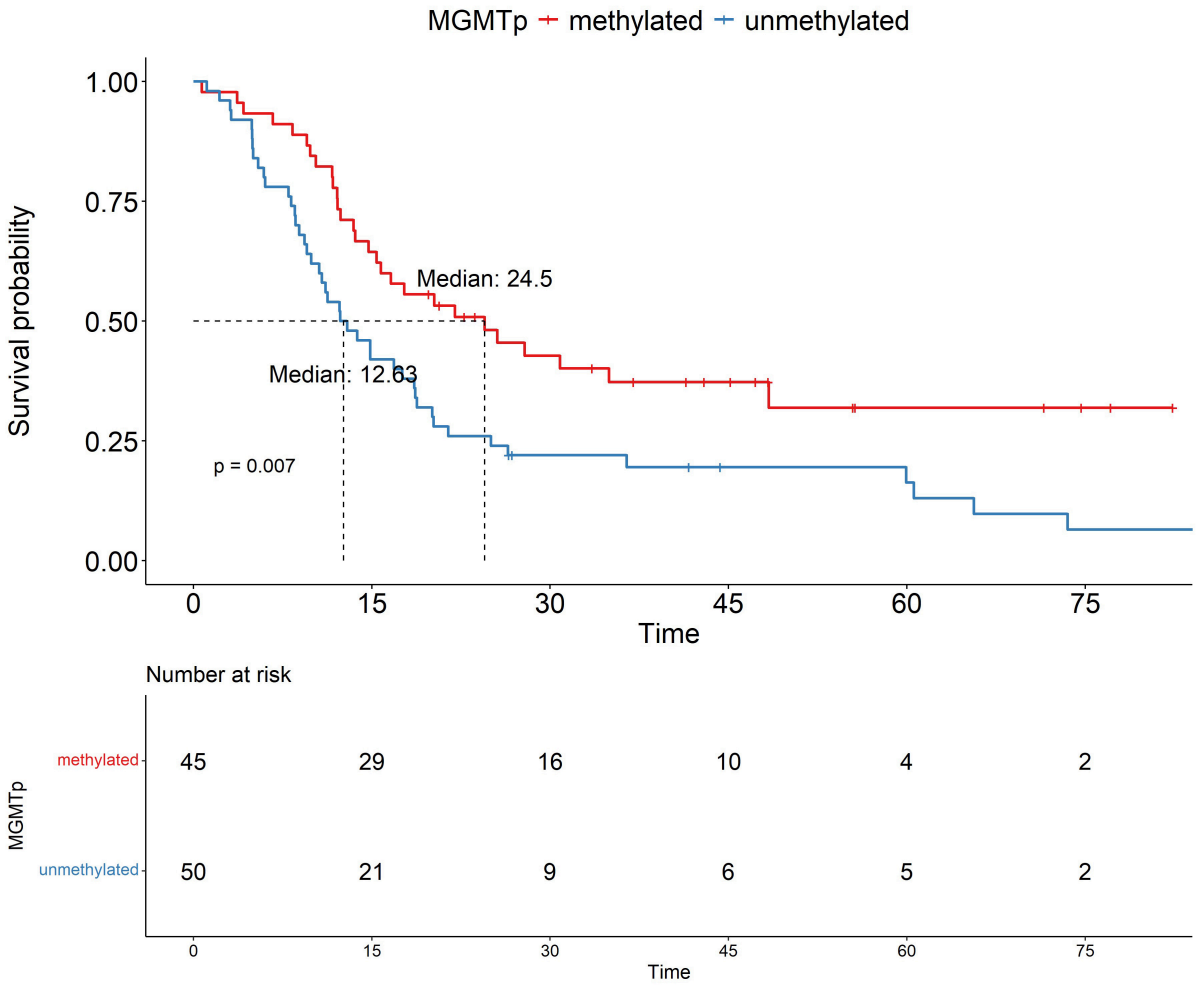
Prognostic impact of MGMTp methylation status. In the GBM patient cohort, OS analysis based on MGMTp methylation status shows that the OS in the unmethylated MGMTp group is poorer (*p* = 0.007).

**Figure 2:**
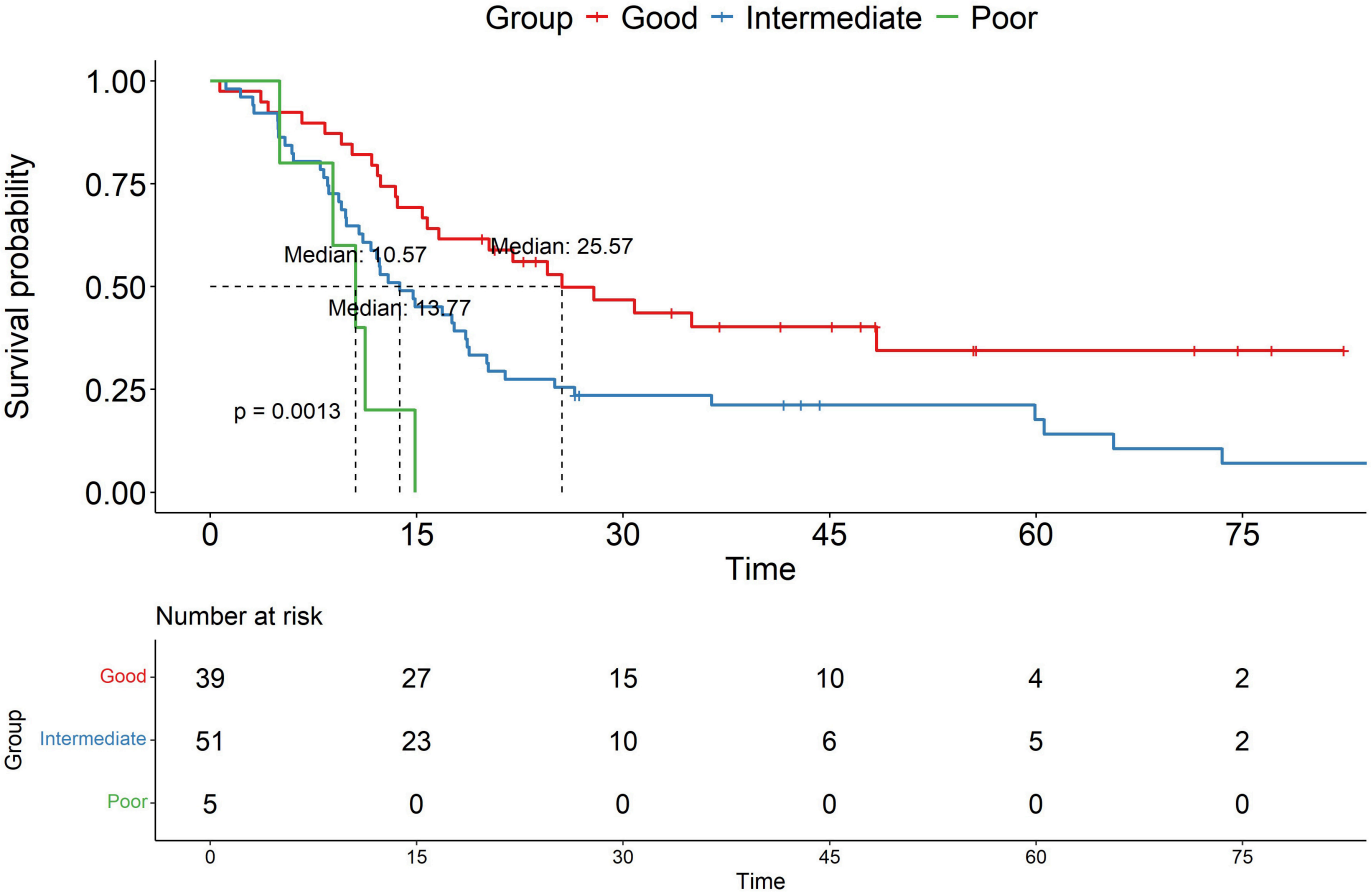
Prognostic impact of *NF1*. OS analysis based on *NF1* status in the same cohort reveals that patients with *NF1* mutation have a poorer OS (*p*= 0.044).

We aimed to identify prognostic factors that negatively impact OS through Kaplan-Meier survival curve analysis. Figure 1 shows the OS plots for two factors that demonstrated associations with poorer OS and were evaluable for Kaplan-Meier analysis. The median OS for the MGMTp(+) and MGMTp(–) groups was 24.5 and 12.63 months, respectively. In the *NF1*(+) and *NF1*(–) groups, the median OS was 18.62 and 11.67 months, respectively (Figure 2). However, given the limited number of NF1(–) cases in our cohort, the survival outcomes regarding *NF1* should be interpreted as an exploratory finding.

### 3.5 Survival outcomes according to combined NF1 and MGMTp status

**Figure 3:**
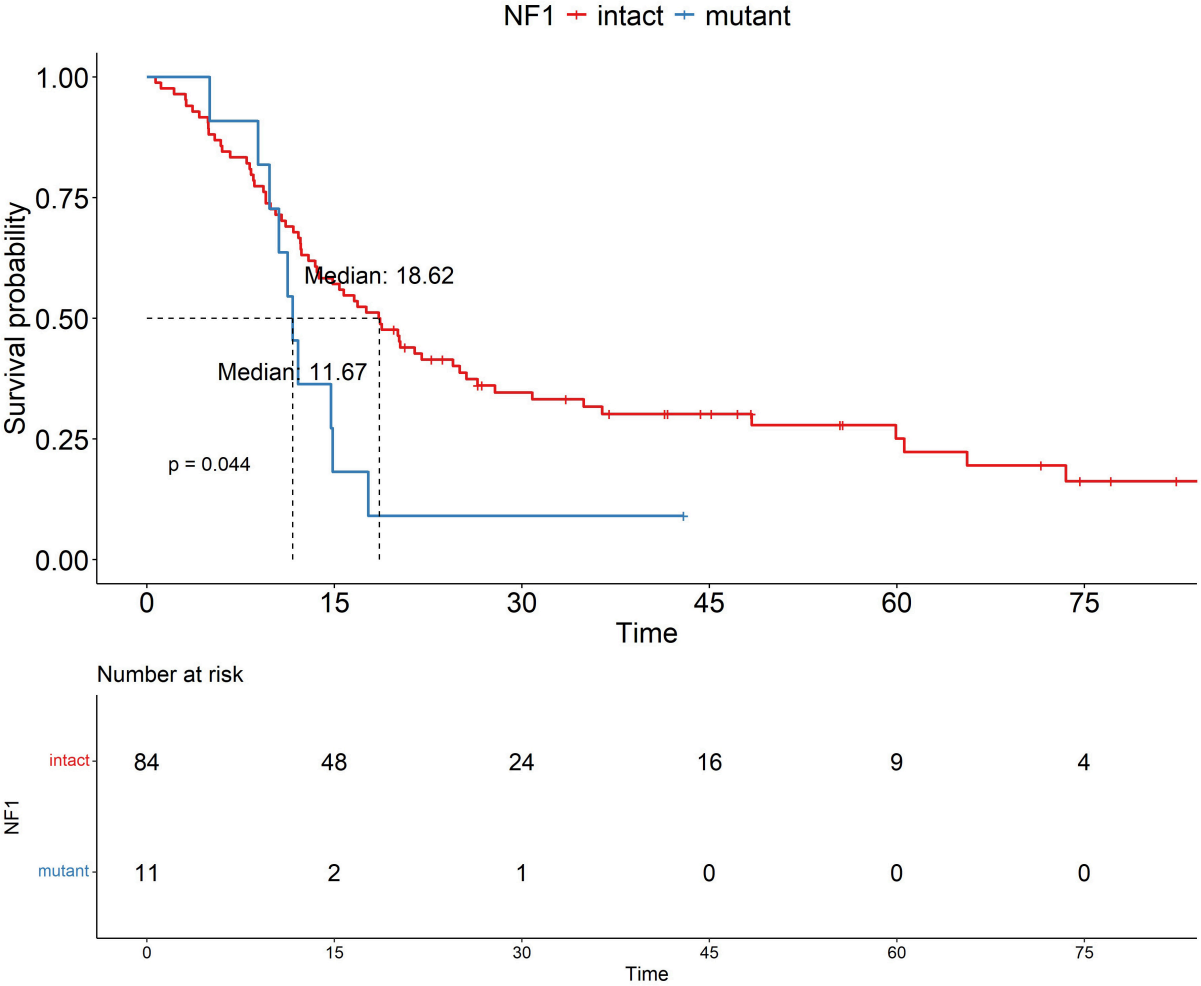
The combined prognostic impact of MGMTp methylation status and *NF1* status in GBM patients. The Poor, Intermediate, and Good groups correspond to unmethylated MGMTp with *NF1* mutation, either unmethylated MGMTp or *NF1* mutation, and methylated MGMTp with intact *NF1*, respectively.

To explore the combined impact of MGMTp and *NF1* status on OS, we stratified the cohort into four subgroups based on their specific genetic combinations: MGMTp(+)/ *NF1*(+) [*n* = 39], MGMTp(+)/ *NF1*(–) [*n* = 6], MGMTp(–)/ *NF1*(+) [*n* = 45], and MGMTp(–)/ *NF1*(–) [*n* = 5]. Subgroup survival analyses were initially conducted to observe variations within each genetic background (Supplementary Figure S1). The most notable OS variation in the log-rank test was observed within the *NF1*(+) group, where survival significantly differed according to MGMTp status (*p* = 0.01). To further evaluate survival differences across these specific combinations, we compared the survival durations of the subgroups (Supplementary Figure S2). While survival differences isolated strictly within the *NF1*(–) background were difficult to assess robustly due to sample size constraints, the co-occurrence of both *NF1*(–) and MGMTp(–) alterations resulted in the most pronounced reduction in OS compared to other combinations. Based on these exploratory observations, we categorized the patients into three risk groups to reflect their combined genetic profiles: a “Good” risk group with both favorable statuses [MGMTp(+) and *NF1*(+)], an “Intermediate” risk group harboring one unfavorable status [either MGMTp(–) or *NF1*(–)], and a “Poor” risk group defined by the co-occurrence of both unfavorable statuses [MGMTp(–) and *NF1*(–)]. Kaplan-Meier analysis revealed significant differences in OS among these three groups (*p* = 0.001, Figure 3). The “Poor” risk group exhibited the shortest median survival (10.57 months), followed by the “Intermediate” (13.77 months) and “Good” groups (25.57 months). However, it should be noted that statistical interpretations of these combined subgroups remain strictly exploratory due to the limited number of cases in the *NF1*(–) subsets (*n* = 5 and *n* = 6) (Figure 3).

## 4 Discussion

In this study, our goal was to identify prognostic factors that adversely affect OS in GBM patients within a Korean cohort. We first examined whether the overall mutational landscape observed in our cohort was consistent with molecular features reported in previous GBM studies. As summarized in Table 2, the most frequently observed alterations in our cohort were detected at frequencies broadly comparable to those reported in previous GBM studies. Furthermore, recurrent copy number amplifications involving *EGFR, PDGFRA, CDK4*, and *KIT* were commonly observed, in line with known genomic profiles of GBM. Minor differences in the relative frequencies of specific alterations may reflect differences in cohort composition, such as restriction to *IDH* wildtype tumors, population-specific characteristics, or technical factors related to NGS panel design and variant classification criteria. Moreover, some previously published studies were conducted prior to the implementation of the 2021 WHO classification, which may contribute to variability in reported mutation frequencies due to differences in tumor classification frameworks. Overall, these findings indicate that the genomic landscape of our cohort is representative of previously reported GBM cohorts, providing an appropriate molecular context for subsequent prognostic analyses.

To maintain statistical rigor and mitigate potential overfitting, we prioritized six core clinical and molecular variables in our multivariable model. This selection process adhered to the proportional hazards assumption and sample size constraints, leading to the exclusion of variables with limited case numbers or those violating statistical assumptions. Consequently, our multivariable analysis suggests that *NF1* mutation may serve as a potential indicator of unfavorable OS in IDH-wildtype GBM patients, even when adjusted for well-established factors such as *MGMT*p methylation status. Although *SETD2* and *PPM1D* mutations showed a potential co-occurrence with *NF1* alterations in our cohort, the small number of cases (*n* = 3) necessitates further validation in larger independent studies. Our observations regarding *NF1* are consistent with the trends reported in existing literature. A study by Wang et al. (11) on the genetic landscape of GBM patients noted that *NF1* mutations could be associated with less favorable survival outcomes. While that study followed the older WHO 2016 classification (CNS 4th edition) rather than the updated 2021 molecular criteria applied in our research, the association between *NF1* alterations and adverse survival remains consistent with our findings. Notably, while Wang et al. primarily reported results from univariate analyses, our study further suggests the potential relevance of *NF1* as a prognostic indicator even after adjusting for key confounders such as initial surgical method, and ECOG in a multivariable context. These findings, taken together, suggest that *NF1* mutation status may provide potential prognostic indicator for *IDH* wildtype GBM patients. In the **OS** analysis, MGMTp unmethylation and *NF1* mutation emerged as potential indicators of shortened survival in this cohort. Consistent with previous studies, the unmethylated MGMTp was again confirmed as a poor prognostic factor (12, 13). Our observation of reduced OS in *NF1* mutation patients is in alignment with the findings of Razis et al. (14). However, the study by Razis et al. (14) used a cohort of 101 patients, including data from Grade 3 glioma patients (Grade 3 glioma: GBM = 16:85). In their multivariate Cox analysis, *NF1* did not show a statistically significant impact. Notably, only variables with a *p*-value < 0.005 from univariate Cox analysis were included in their multivariate model. This likely obscured the impact of *NF1*, as the analysis was restricted to variables with very strong effects (15). Although the prognostic impact of *NF1* mutation has not been consistently demonstrated across all cohorts, its potential risk has been repeatedly suggested in multiple cohort studies and case reports (11, 14, 16). Our stratified survival analysis (Supplementary Figure S2) suggests that *NF1* mutations may act synergistically with MGMTp unmethylation to worsen prognosis. However, a formal multivariable Cox interaction analysis (*NF1* × MGMTp) did not reach statistical significance (*p* = 0.784). This lack of significance is likely attributable to insufficient statistical power, given the extremely small number of patients (*n* = 5) harboring both *NF1* mutations and unmethylated MGMTp in the multivariable model. Therefore, it would be premature to define *NF1* mutation as an established, independent prognostic factor based solely on the current findings. Instead, its prognostic utility should be carefully interpreted as a context-dependent indicator that complements MGMTp status. A more definitive assessment of the role of *NF1* will require larger patient cohorts with a higher prevalence of *NF1* alterations to ensure sufficient statistical power. Taken together, these findings suggest that the *NF1* mutation may provide additional prognostic context in specific GBM cohorts, although its definitive role as an established prognostic marker remains to be validated in larger and independent datasets.

The neurofibromin protein expressed by *NF1* acts as a tumor suppressor gene and is a regulator of the *RAS/MAPK* pathway, primarily expressed in the central nervous system (17, 18). In *NF1*-deficient cells, sustained activation of the *RAS/RAF/MAPK* pathway excessively promotes cell proliferation. Additionally, the *PI3K/AKT/mTOR* pathway is constitutively activated, disrupting apoptotic signaling and inhibiting the apoptosis of abnormal cells, thereby contributing to tumorigenesis (19). However, it should be noted that our current study lacks direct molecular data to confirm these specific pathway activations within our cohort, and this mechanistic rationale remains theoretical based on existing literature. Although studies and case reports have been conducted on the impact of *NF1* in GBM patients, the precise mechanism has not yet been clearly elucidated. Consistent with the scope of these previous clinical case studies, our current research relies on retrospective genomic profiling and does not provide direct functional validation of these downstream pathways. Therefore, while our survival data align with the known mechanistic vulnerabilities of *NF1* deficiency, we conservatively interpret *NF1* mutation status primarily as a prognostic biomarker for risk stratification, rather than drawing definitive functional conclusions. Studies have been conducted across various cohorts to identify recurrent mutation sites or examine the mutation spectrum of *NF1* (20–22). Table 5 lists the *NF1* mutation information obtained from our cohort. Four novel *NF1* mutations were identified (c.2917_2926dup, c.3188_3195delGTCTTACA, c.5827delC, c.7512_7522delTGCAGCCACCT). When compared with the TCGA GBM cohort, two *NF1* mutations were found at the same locations (c.4600C>T, c.499_502delGTTT). Further studies are warranted to investigate the association between *NF1* mutations at these locations and the development of GBM. Notably, the same mutation location (c.4600C>T) was identified in a study on *NF1* patients in Korea (20). However, most studies have focused on recurrent *NF1* mutations rather than investigating *NF1* mutations specifically within GBM patient populations, making it challenging to identify *NF1* mutation hotspots that contribute specifically to GBM development.

**Table 5:** List of NF1 mutations obtained from this study.

Patients_id	Age	Amino acid mutation	Nucleotide mutations	OS	Reference
4	81	p.His1943ThrfsTer7	c.5827delC	14.7	This report
33	89	p.Arg1534Ter	c.4600C>T	5.0	Side et al. (34)
45	55	p.Leu1253fs	c.3758_3762delTCTAC	14.9	Fahsold et al. (35)
47	70	p.Ala2505fs	c.7512_7522delTGCAGCCACCT	12.1	This report
47	70	p.Arg2637Ter	c.7909C>T	12.1	Upadhyaya et al. (36)
57	48	p.Phe1247IlefsTer18	c.3739_3742delTTTG	8.9	Fahsold et al. (35)
60	64	p.Ile679AspfsTer21	c.2033_2034insC	11.3	Heim et al. (37)
60	64	p.Glu2195LeufsTer4	c.6583_6586delGAGA	11.3	Fahsold et al. (35)
66	87	p.Cys167fs	c.499_502delGTTT	9.8	Upadhyaya et al. (38)
95	57	p.Trp696Ter	c.2087G>A	42.9	Fahsold et al. (35)
103	86	p.Gln1724Ter	c.5170C>T	11.7	Fahsold et al. (35)
124	50	p.Gly823Ter	c.2467G>T	17.7	Fahsold et al. (35)
124	50	p.Thr976ArgfsTer2	c.2917_2926dup	17.7	This report
128	70	p.Cys1063Ter	c.3188_3195delGTCTTACA	10.6	This report

## 5 Limitations

This study has several limitations.

First, although the targeted NGS panel used in this study is capable of detecting a broad range of variant types, all *NF1* alterations identified in this cohort were truncating mutations (frameshift or nonsense). Therefore, the prognostic analyses primarily reflect the impact of loss-of-function *NF1* mutations, and the findings may not be generalizable to glioblastoma cases harboring *NF1* missense variants or other non-truncating alterations.

Second, this study was conducted as a single-center retrospective analysis with a relatively limited sample size, which may reduce statistical power and limit the generalizability of the results. Larger, multi-center studies will be required to validate the prognostic significance of *NF1* alterations in glioblastoma.

Third, our analysis of treatment impact was restricted by the retrospective nature of the data. While we incorporated the initial surgical method to adjust for baseline surgical intervention, this binary variable cannot fully substitute for precise volumetric extent of resection. Furthermore, detailed information regarding the completion rates of radiotherapy and variations in temozolomide administration could not be fully captured, which may introduce residual confounding regarding survival outcomes.

## 6 Conclusion

Our study suggests that *NF1* mutation may be associated with poorer survival in *IDH*-wildtype GBM patients within a Korean cohort. While *NF1* mutation was associated with survival outcomes in multivariable Cox analysis, its impact appears to be context-dependent, showing a complementary prognostic value when integrated with MGMTp methylation status. These findings indicate that *NF1* alterations may provide meaningful prognostic context in glioblastoma and highlight the potential value of integrated molecular characterization for refined risk stratification. Further studies in larger and independent cohorts are warranted to definitively establish the prognostic role of *NF1* across diverse patient populations.

## Data Availability

The data analyzed in this study is subject to the following licenses/restrictions: The data used in this study were obtained from clinical patients and therefore cannot be made publicly available. Requests to access these datasets should be directed to kysrosa@hanmail.net.
