# Supplementary material for: Prognostic impact of NF1 mutation in Korean cohort with glioblastoma
Source: Front Neurol. 2026 May 21;17:1752936. doi: 10.3389/fneur.2026.1752936 (PMC13233182; doi:10.3389/fneur.2026.1752936)
Supplement: Supplementary file 1 [file Data_Sheet_1.pdf]

Supplementary Material

Title : Prognostic Impact of *NF1* Mutation in Korean Cohort with Glioblastoma

Ryong Heo[a,¶], Yeosong Kim[b,¶], Sehyeon Kim[a], Boseong Kim[c], Sinsoo Jeun[d] and Chihyun Park[a,e,*]

[a]Department of Data Science, Kangwon National University, Chuncheon-si, 24341, Gangwon-do, Republic of Korea, [b]Departments of Neurosurgery, GSAM Hospital, Gunpo, Republic of Korea, [c]Department of Biotechnology and Bioengineering, Kangwon National University, Chuncheon-si, 24341, Gangwon-do, Republic of Korea, [d]Department of Neurosurgery, Seoul St. Mary's Hospital, College of Medicine, The Catholic University of Korea, Seoul, Korea, [e]Department of Computer Science and Engineering, Kangwon National University, Chuncheon-si, 24341, Gangwon-do, Republic of Korea

***Corresponding Authors**

E-mail : chihyun@kangwon.ac.kr

[¶]These authors contributed equally to this work.

Supplementary Methods S1. Integrated Diagnostic Workflow for Glioblastoma According to WHO CNS5

All cases included in this study were diagnosed using an integrated diagnostic framework in accordance with the 2021 World Health Organization (WHO) Classification of Tumors of the Central Nervous System (CNS5). The final diagnosis was rendered on a case-by-case basis through combined interpretation of histopathological features, immunohistochemistry, and molecular analyses performed as part of routine clinical practice.

Histopathological Evaluation

All specimens underwent intraoperative frozen-section examination followed by definitive histopathological assessment of permanent sections by board-certified neuropathologists. Histological features characteristic of glioblastoma, including high cellularity, mitotic activity, microvascular proliferation, and/or necrosis, were evaluated in accordance with WHO CNS5 criteria.

Immunohistochemical Analyses

Immunohistochemistry (IHC) was performed to support lineage determination and to exclude alternative diagnoses. The following markers were evaluated as part of the diagnostic workup:

- GFAP and Olig2, to support glial lineage
- IDH1 (R132H), to screen for IDH-mutant gliomas
- ATRX and p53, to aid in molecular subtype inference and differential diagnosis
- Synaptophysin, to exclude neuronal tumors
- EMA, to exclude epithelial or meningioma-like tumors
- Ki-67, to assess proliferative activity
- H3 K27M, when clinically indicated, to evaluate H3-altered gliomas

These IHC results were used as supportive diagnostic information and were not interpreted in isolation.

Molecular Analyses Using Targeted NGS Panels

Targeted next-generation sequencing (NGS) was performed using the Oncomine Comprehensive Assay Plus (Thermo Fisher Scientific) to evaluate clinically relevant genomic alterations. Library preparation and sequencing were conducted using the Ion Chef™ system and Ion S5™ XL Sequencer (Thermo Fisher Scientific).

- Reference genome: hg19
- Analysis pipeline: Torrent Suite v5.10.2 → Ion Reporter v5.12 → Oncomine Knowledgebase Reporter v4.7

The NGS panel was used to assess hotspot mutations and copy number alterations involving key genes relevant to glioblastoma diagnosis and classification, including IDH1, IDH2, EGFR, and TERT promoter.

Variant Detection and CNV Calling Criteria

Variant calling and copy number variation (CNV) analyses were performed using the following predefined thresholds:

- Minimum allele frequency for hotspot variants: ≥ 4%
- Minimum allele frequency for indel variants: ≥ 5%
- Minimum allele frequency for SNP variants: ≥ 5%
- Minimum read counts for gene fusions: ≥ 40
- CNV gain threshold: copy number ≥ 4
- Gain confidence level: 0.05
- Maximum fold difference for CNV loss: 0.7

Using these criteria, EGFR amplification and IDH2 copy number gains were explicitly assessed at the genomic level. Importantly, EGFR protein expression evaluated by immunohistochemistry was not used as a surrogate for gene amplification.

Role of FISH Analyses

Fluorescence in situ hybridization (FISH) analyses for 1p36 and 19q13 deletions were performed in selected cases as part of the routine differential diagnostic workup to exclude oligodendroglioma, as 1p/19q codeletion is a defining molecular feature of oligodendroglioma but is not a diagnostic criterion for glioblastoma under the WHO CNS5 classification.

In addition, chromosome 7 gain and chromosome 10 loss were evaluated by board-certified neuropathologists using available cytogenetic and molecular pathology data, including FISH-based assessments when applicable. The presence of chromosome 7 gain and chromosome 10 loss, a molecular signature relevant to the WHO CNS5 definition of glioblastoma, was incorporated into the integrated diagnostic decision-making process.

These cytogenetic findings were interpreted in conjunction with histopathological features and other molecular results and were not considered in isolation.

Integrated Diagnostic Interpretation

All diagnostic information—including histopathological findings (such as microvascular proliferation and necrosis), immunohistochemical results, targeted NGS-based molecular analyses, and cytogenetic evaluation of chromosome 7 gain and chromosome 10 loss by neuropathologists—was integrated on a case-by-case basis in accordance with the WHO CNS5 classification framework.

Based on this integrated interpretation, all cases were ultimately diagnosed as glioblastoma, IDH-wildtype (CNS WHO grade 4) by experienced neuropathologists.

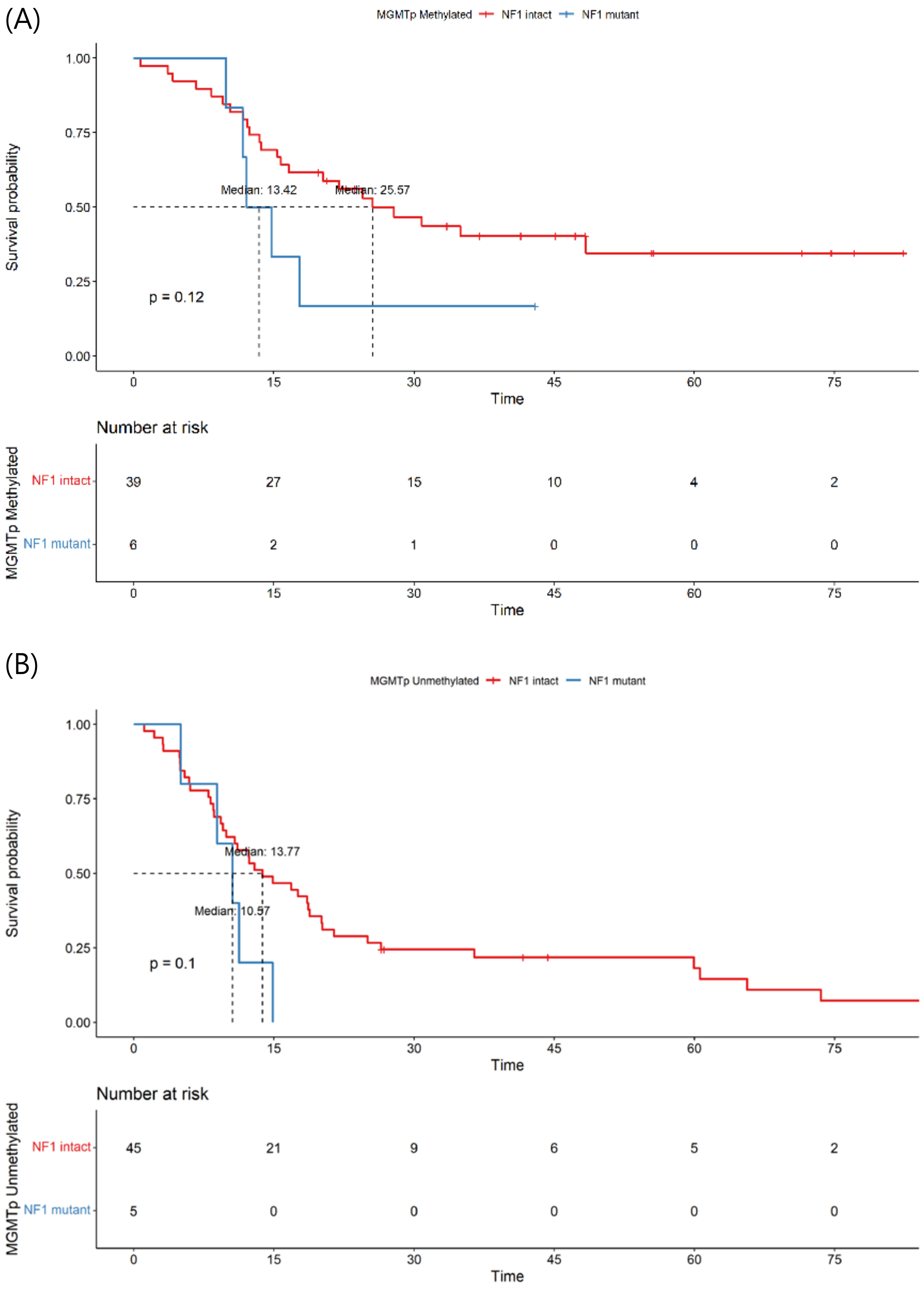
(A)
MGMTp Methylated
NF1 intact
NF1 mutant
Survival probability
1.00
0.75
0.50
0.25
0.00
Median: 13.42
Median: 25.57
p = 0.12
0
15
30
45
60
75
Time
Number at risk
MGMTp Methylated
NF1 intact 39 27 15 10 4 2
NF1 mutant 6 2 1 0 0 0
(B)
MGMTp Unmethylated
NF1 intact
NF1 mutant
Survival probability
Median: 13.77
Median: 10.57
p = 0.1
Time
Number at risk
MGMTp Unmethylated
NF1 intact 45 21 9 6 5 2
NF1 mutant 5 0 0 0 0 0

Supplementary Figure S1

(C)

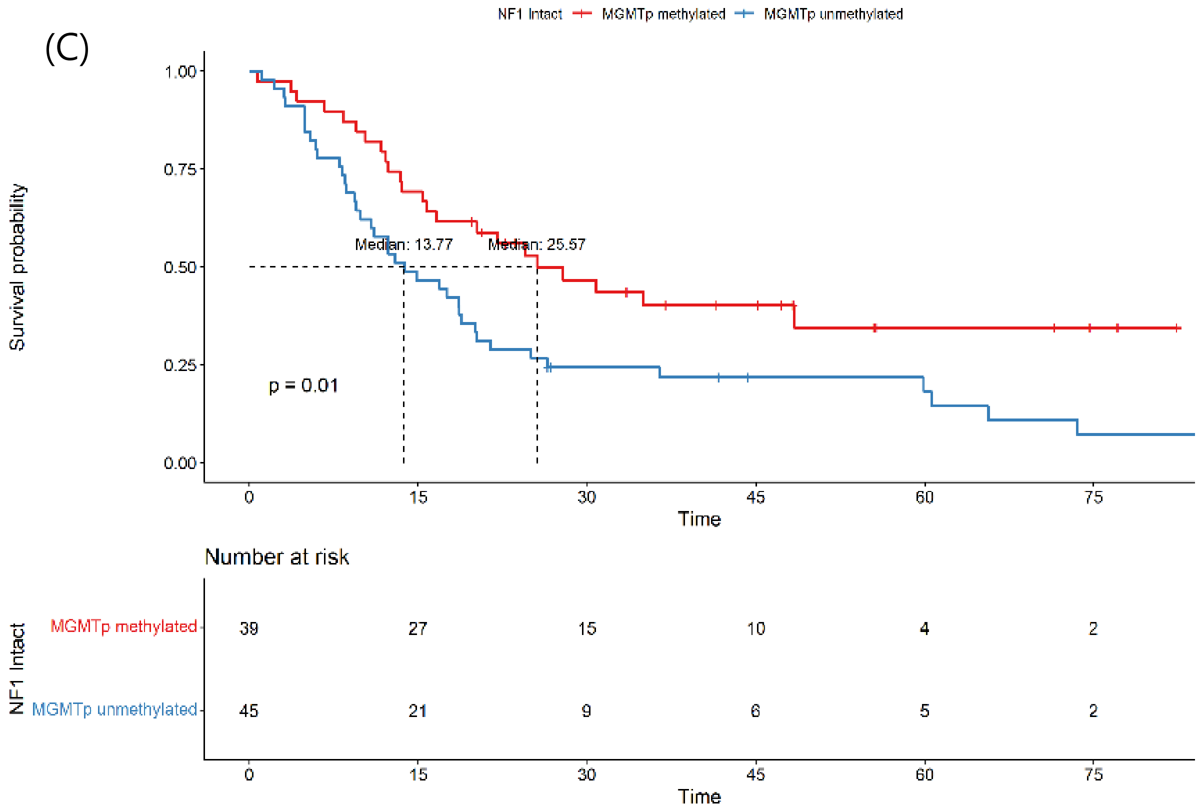

(D)

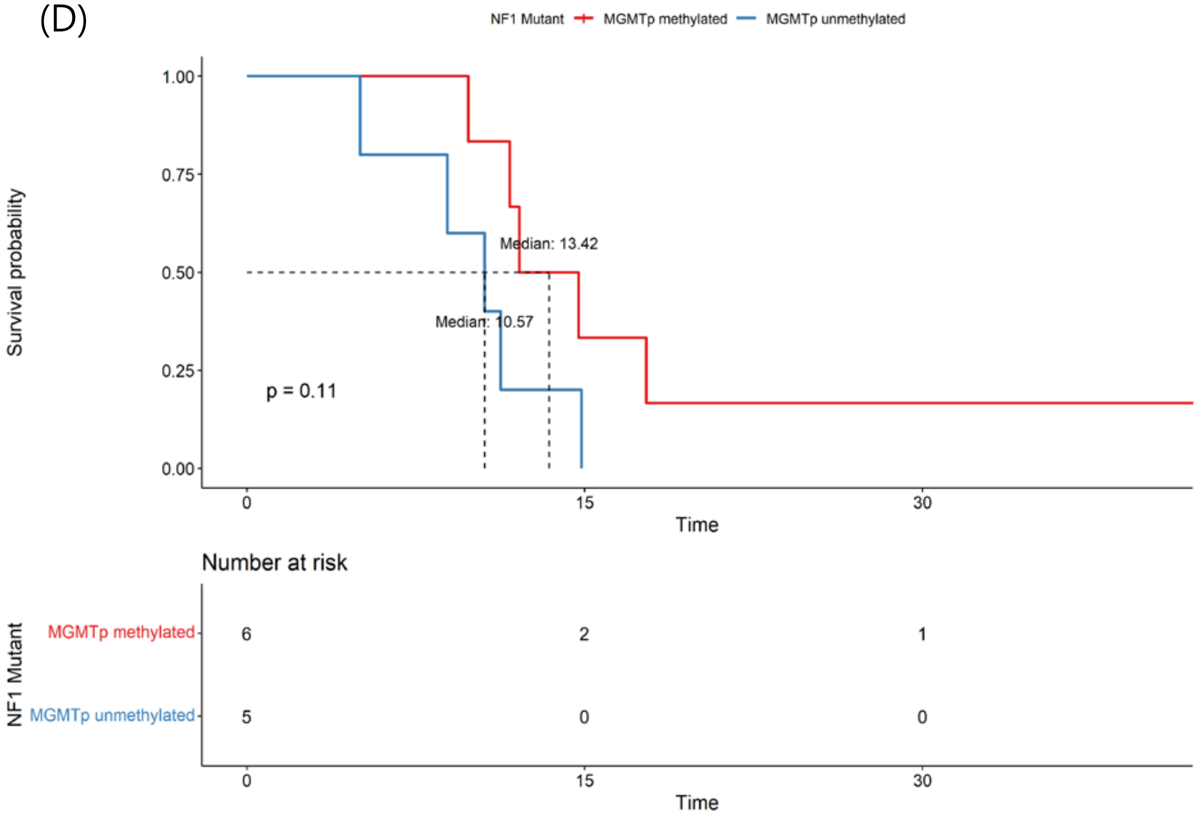

(A) In the *MGMTp*-methylated group (n=45), the median OS for the *NF1*-intact and *NF1*-mutant groups was 25.57 and 13.42 months, respectively. (B) In the *MGMTp*-unmethylated group (n=50), the

median OS was 13.77 months for the *NF1*-intact group and 10.57 months for the *NF1*-mutant group. (C) In the *NF1*-intact group (n=84), the median OS for the *MGMTp*-methylated and -unmethylated groups was 25.57 and 13.77 months, respectively. (D) Among *NF1*-mutant patients (n=11), the median OS was 13.42 months in the *MGMTp*-methylated group and 10.57 months in the unmethylated group. Of these, only the comparison in (C) showed a significant difference by log-rank test, with *MGMTp* methylation status impacting OS in the *NF1*-intact group. Taken together, these findings suggest that *NF1* mutation does not show as pronounced an impact on OS when *MGMTp* methylation status is concurrently considered. However, the p-values of 0.12 and 0.10 in (A) and (B) respectively indicate a minor impact of *NF1* status on OS, even when *MGMTp* status is accounted for.

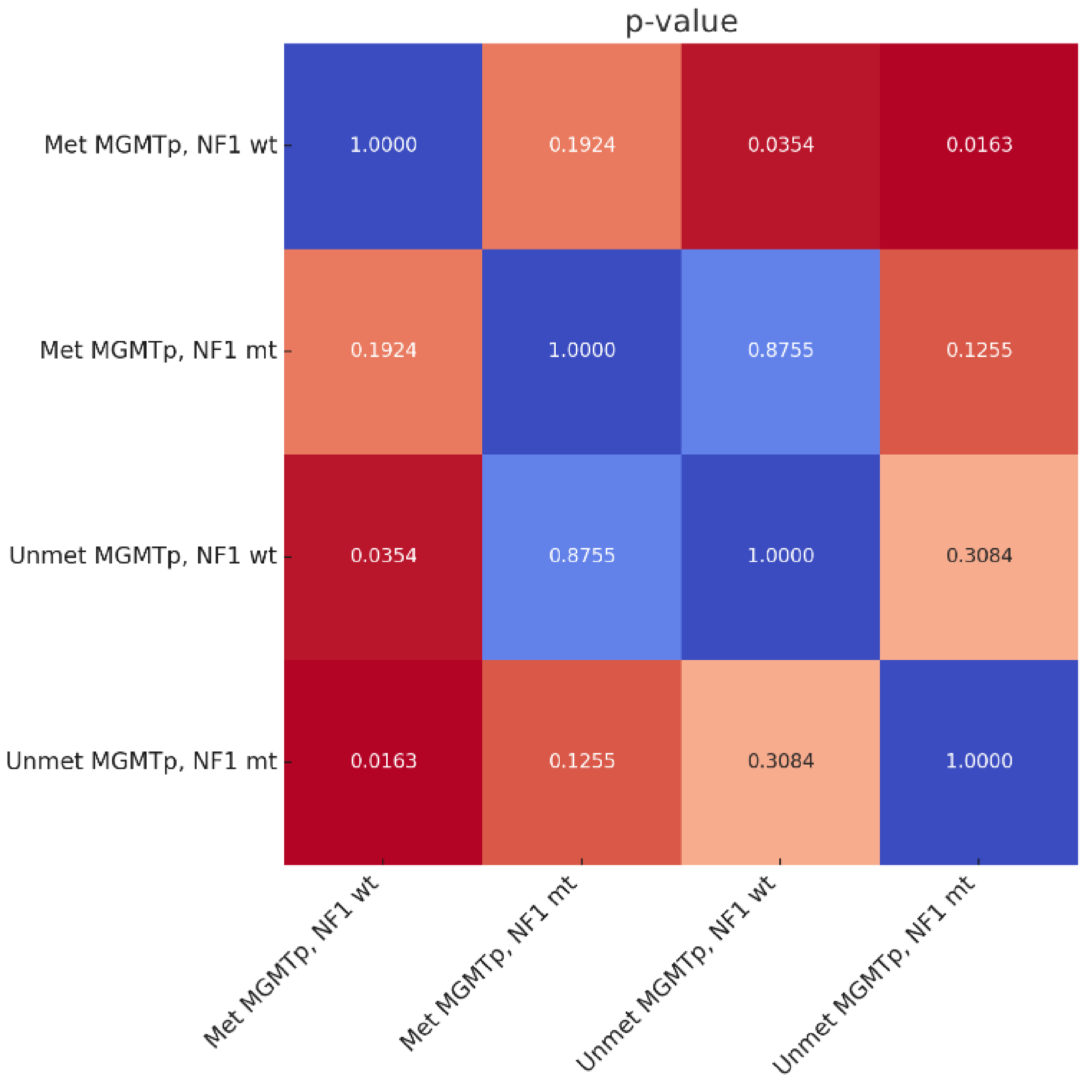

Supplementary Figure S2

This heatmap illustrates the p-values from U-tests conducted across OS values in the four defined groups. A significant difference was observed between the *MGMTp*(-) with *NF1*(+) and *MGMTp*(+) with *NF1*(+) groups (p=0.035), indicating that *MGMTp* methylation status significantly impacts OS within the *NF1*(+) group (n=84). Conversely, *MGMTp* methylation status did not show a statistically significant effect in the *NF1*(-) group (n=11; p=0.125). *NF1* status itself was not significant within either the *MGMTp*(+) or *MGMTp*(-) groups (p=0.192 and p=0.308, respectively). The lowest p-value was observed between the *MGMTp*(-) with *NF1*(+) and *MGMTp*(+) with *NF1*(-) groups (p=0.016), suggesting that while *NF1*(-) may have a weaker impact on OS compared to *MGMTp* methylation, it acts synergistically with *MGMTp*(-) as a poor prognostic factor.
